# Genetic Diversity and Potential of Cyanobacteria and Fungi Living on Arctic Liverworts

**DOI:** 10.1007/s00248-025-02589-y

**Published:** 2025-08-26

**Authors:** Ekaterina Pushkareva, Leonie Keilholz, Justin Böse, Karl-Heinz Linne von Berg

**Affiliations:** Department of Biology, Botanical Institute, University of Cologne, Zulpicher Str. 47B, 50674 Cologne, Germany

**Keywords:** Liverworts, Arctic, Fungi, Cyanobacteria, Metagenomics

## Abstract

Liverworts often form symbiotic associations with fungi and cyanobacteria, yet the distribution and specificity of these relationships remain largely unexplored, particularly in Arctic environments. This study used metagenomic sequencing to investigate fungal and cyanobacterial communities associated with Arctic liverworts, analyzing photosynthetic parts of gametophytes and their rhizoids with attached soil separately. The results revealed that Ascomycota dominated the fungal community. The cyanobacterial community was primarily composed of heterocytous *Nostoc* and non-heterocytous filamentous *Leptolyngbya*, with *Nostoc* showing evidence of nitrogen fixation, especially in gametophytes, suggesting a potential role in enhancing nitrogen availability for the host. These findings underscore the ecological significance of liverwort-associated microorganisms in Arctic ecosystems, with microbial composition differing between upper and lower parts of plants, as well as between leafy and thalloid liverworts, indicating possible functional specialization.

**Supplementary Information:**

The online version contains supplementary material available at 10.1007/s00248-025-02589-y.

Among other mosses, liverworts represent ecologically significant components of Arctic ecosystems, where extreme cold, short growing seasons, and nutrient limitations pose considerable challenges to plant survival. They often form relationships with fungi and cyanobacteria, which may enhance their ability to withstand harsh conditions [1]. These relationships are mutually beneficial, with microorganisms providing nutrients to the liverworts in return for carbohydrates and lipids [2]. Some studies suggest physical associations such as fungal colonization of thalli or rhizoids and intracellular colonization by cyanobacteria in specialized structures, while in other cases, co-occurrence may reflect epiphytic or rhizospheric relationships rather than true symbiosis [3, 4]. However, the nature, diversity, and ecological significance of these associations in the liverworts from high Arctic remain underexplored. The objective of this study was to investigate the fungal and cyanobacterial communities associated with Arctic liverworts. We hypothesized that fungal communities will be more abundant and diverse in the rhizoid area including surrounding soil, facilitating nutrient and water uptake, whereas cyanobacteria will be more prevalent in the upper photosynthetic part of the plant, supporting nitrogen fixation. Furthermore, differences in microbial composition between both tissues were expected to reflect functional specialization, thus providing insights into the ecological roles of these associations.

Four liverworts were collected in July 2023 in Ny-Ålesund and Longyearbyen (Svalbard, high Arctic), respectively (Table 1). Morphological identification was conducted using the standard taxonomic keys [5] and microscopy techniques. The upper part (photosynthetic gametophyte) and the lower part (containing rhizoids and surrounding soil) were separated in the lab within 24 h of collection under a dissecting microscope using sterile tools. However, due to the delicate, single-celled nature of liverwort rhizoids, it is possible that some rhizoids remained attached to both the upper and lower parts, making a complete separation difficult. In addition, the upper part of the liverworts was gently rinsed with sterile distilled water to remove loosely attached microorganisms.

**Table 1:** Description of the collected liverworts

Sample	Location	GPS	Altitude, m a.s.l	Top hit	Type of liverwort
Liv1	Longyearbyen	N78.20138, E15.57381	115	*Cephalozia bicuspidata*	Leafy
Liv2	Ny-Ålesund	N78.93864, E11.78892	43	*Ptilidium ciliare*	Leafy
Liv3		N78.97935, E12.07709	278	*Sauteria alpina*	Thalloid
Liv4		N78.95027, E12.47808	102	*Aneura pinguis*	Thalloid

DNA extraction from the lower and upper parts of collected liverworts was performed using the DNeasy PowerSoil Pro Kit (QIAGEN, USA) and Plant Kit, respectively. The extracted DNAs were sent to Eurofins Genomics (Germany), where metagenomic sequencing on an Illumina NovaSeq 6000 S4 (PE150) was conducted. The raw reads were submitted to the Sequence Read Archive (SRA) under the project PRJNA1257069.

Bioinformatic analysis was performed in the OmicsBox software (Biobam, Spain). Quality filtering of the reads was conducted in Trimmomatic [6], resulting in 542 M reads in total. The taxonomic assignment of the 16S (for cyanobacteria) and 18S (for liverworts) rRNA reads was performed using the Silva database (v. 138.2). ITS reads were used for the fungal classification with the UNITE database (v. 10.0). In addition, cyanobacteria were classified into three morphological groups based on their growth forms: filamentous, heterocytous, and unicellular [7]. Similarly, fungi were assigned to functional guilds according to their primary ecological roles using the FungalTraits database [8]. The functional potential of the most dominant genus *Nostoc* associated with liverworts was studied by extracting sequences from selected genera using the Kraken2 database (v. 2.1.3). The extracted reads were assembled de novo using MEGAHIT (v. 1.2.8; [9]) and quantified as fragments per kilobase of contig per million fragments sequenced (FPKM; [10]). Further, the contigs were aligned to the NCBI BLAST searches (E × 10) and Gene Ontology (GO; [11]). Additionally, functional annotation of the whole assembly was conducted using EggNOG [12].

One-way and two-way analyses of variance (ANOVA) and subsequent Tukey’s HSD post hoc test (*p*-value < 0.05) were carried out in R (v. 4.2.1). Genera with a low number of reads were excluded from the analysis. Prior to analysis, normality of variance was assessed using Shapiro–Wilk’s test, and data were SQRT transformed, if necessary.

The collected liverworts were identified as *Cephalozia bicuspidata* (Liv1), *Ptilidium ciliare* (Liv2), *Sauteria alpina* (Liv3), and *Aneura pinguis* (Liv4) using both morphological and molecular methods. All four liverworts have previously been reported in Svalbard [13].

Metagenomic analysis produced 473 K ITS and 2 K rRNA reads assigned as fungi and cyanobacteria, respectively. The majority of the recorded fungi belonged to the phylum Ascomycota, with a significantly higher prevalence in the upper part (Suppl. Table 1). Ascomycota has previously been recorded to form mycothalli on Arctic liverworts, such as Anastrophyllaceae, Cephaloziellaceae, and Cephaloziaceae [14]. Similarly, most of the other fungal phyla showed a higher number of reads in the upper parts of the plants, but no significant differences were observed between the two liverwort growth forms (leafy vs. thalloid). The functional classification of fungi showed the dominance of saprotrophic traits across all samples (Fig. 1a, Suppl. Table 2). Liverworts frequently occur in moist environments where organic material, including decaying plant matter, accumulates, thereby providing a substrate for saprotrophic fungi [15]. Parasitic and pathogenic fungi also constituted substantial proportions in the majority of the samples. While these fungi are typically known to have a negative impact on plants, some have been observed to exhibit a more dynamic interaction, shifting between parasitic and beneficial behaviors depending on the stage of their life cycle [16]. Furthermore, ectomycorrhizal (ECM) fungi were found to be highly abundant and significantly more prevalent on the upper parts of liverworts. Although ECM fungi are traditionally associated with vascular plants, their presence on liverworts suggests a potentially important but understudied symbiotic relationship [17]. The symbiotic interactions involving ECMs have played a pivotal role in the evolution and ecological success of land plants by facilitating nutrient acquisition and stress tolerance [18]. This underscores the possibility that early diverging plants, such as liverworts, may also engage in these beneficial associations. In addition, arbuscular mycorrhizal fungi (AMF), which often form mutualistic associations with liverworts [19], were also present and dominated the upper parts of the plants, confirming the importance of AMF–liverwort symbiosis.

**Fig. 1:**
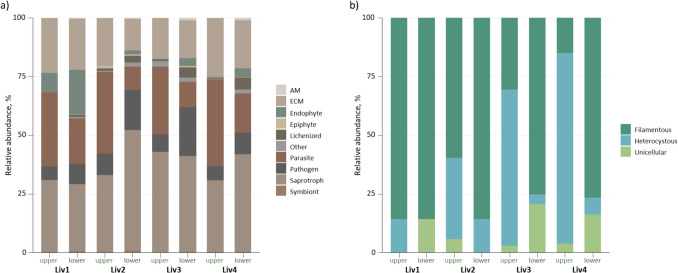
Relative abundance of fungal traits **a** and cyanobacterial forms **b** recorded in the studied liverworts (upper and lower correspond to different parts of liverwort)

Interestingly, *Tulasnella*, which has been documented to form ectomycorrhizal associations and even act as fungal partners in epiparasitic relationships with the liverwort *Cryptothallus mirabilis* (*Aneura mirabilis*) [20], had a high number of reads within the thallus of *Sauteria alpina* (Liv3). This might suggest that similar specialized interactions could occur between these two organisms, potentially indicating a complex ecological role where the liverwort may engage in nutrient exchange or even parasitic relationships mediated by these fungi. Similarly, the ascomycete fungus *Pezoloma* was detected primarily in *Cephalozia bicuspidata* (Liv1). *Pezoloma* species are known to form ericoid mycorrhizas, and their interaction with *C. bicuspidata* has been shown to involve bidirectional nutrient exchange, resulting in enhanced liverwort growth [21]. Furthermore, *Cortinarius* and *Suillus* (ECM fungi) were among the most dominant genera detected in the samples. Their presence was likely due to spores or inactive DNA, and their higher abundance in the upper parts of the liverworts may reflect aerial deposition rather than active colonization. In addition, two parasitic genera (*Gromochytrium* and *Phlyctochytrium*) and one pathogenic genus (*Pseudocercospora*) exhibited high read numbers, with significant prevalence in the upper parts across all studied liverworts. While these fungi are known to harm vascular plants (e.g., [22]), there is currently no information available regarding their effects on liverworts.

Cyanobacteria have been well-documented as epiphytes on numerous hepatics and were detected in all samples. However, *C. bicuspidata* (Liv1) had a low cyanobacterial abundance (< 0.1% of all bacterial reads). In contrast, three other studied liverworts (Liv2, 3, and 4) exhibited a greater proportion of cyanobacteria (1–9% of all bacterial reads). Furthermore, a distinct pattern of cyanobacterial abundance was observed, with higher abundance in the upper part of these three liverworts (Suppl. Table 3). Specifically, heterocytous cyanobacteria dominated the gametophytes of thalloid liverworts, while non-heterocytous filamentous cyanobacteria were more prevalent across both parts of leafy liverworts, as well as the lower part of thalloid liverworts (Fig. 1b). Filamentous cyanobacteria are known to dominate various soil ecosystems, which likely explains their prevalence in rhizoid-soil-associated microbial communities. The high presence of filamentous cyanobacteria on the gametophyte may be attributed to their ability to adhere to moist surfaces and exploit the nutrient-rich microenvironment provided by the liverwort. Furthermore, the presence of heterocytous cyanobacteria from the order Nostocales, particularly from the genus *Nostoc* (Table 2), is consistent with prior studies documenting their role as epi- or endophytes on Marchantiophyta [23].

**Table 2:** Metagenomic profile of cyanobacteria *Nostoc*

	Liv1		Liv2		Liv3		Liv4	
	upper part	lower part	upper part	lower part	upper part	lower part	upper part	lower part
Number of rRNA reads	1	0	28	3	146	1	655	4
% of cyanobacterial rRNA reads	14.3	0.0	26.9	14.3	44.4	1.1	83.9	4.2
Number of non-rRNA reads	125	14	12100	29	242292	524	11611	1093
nifB	0	0	54	0	887	1	37	7
nifD	0	0	1	0	7	1	3	0
nifE	0	0	36	0	882	6	43	3
nifH	1	0	10	0	351	4	25	4
nifK	0	0	2	0	18	0	3	0
nifN	0	0	4	0	8	0	1	0
nifS	0	0	10	0	5	0	9	0
nifU	0	0	0	0	3	0	0	0
VnfD	0	0	12	0	310	0	24	3
AmtB	0	0	9	0	1	0	1	0
PstB	0	0	35	0	1006	1	25	2

*Nostoc*, associated with liverworts, likely contributes to nitrogen fixation, benefiting the host liverwort by enhancing nutrient availability in nutrient-poor environments [24]. It is also previously shown that bryophytes are rather independent of soil-N resources [2], which explains their higher abundance on the upper part of liverworts (Table 2). Although this study did not directly investigate symbiotic relationships or the influence of environmental factors (e.g., light), the observed cyanobacterial distribution might suggest potential symbiotic associations localized to the upper parts of the plants, possibly reflecting ecological preferences or microenvironmental conditions. Furthermore, the nifH, nifD, and nifK genes are considered the primary genes in nitrogen fixation by heterocytous cyanobacteria, encoding the core components of the Mo-dependent nitrogenase ([25], Suppl. Table 4). However, VnfD, a gene encoding vanadium (V)-nitrogenase, has a comparable abundance to nifH in the three liverworts (Liv2, 3, 4). Vnf genes have previously been identified in *Nostoc* associated with lichens and plants [26], and their presence could serve as a marker gene for *Nostoc*-liverwort symbiosis. The presence of different nitrogenase systems suggests metabolic flexibility, which is crucial for *Nostoc*’s adaptation to various environmental conditions. Furthermore, three liverworts (Liv2, 3, and 4) had a higher abundance of nifB and nifE within nitrogenase structural genes. Some cyanobacteria underwent genome expansion [27], resulting in gene duplication of nifB and nifE genes in *Nostoc*, which may also be the case in the investigated associations.

In addition, KEGG pathway analysis revealed that functional differentiation of microbial communities was primarily driven by liverwort growth form (thalloid vs. leafy; Suppl. Table 5). Pathways involved in central carbon metabolism (e.g., citrate cycle, propanoate, and butanoate metabolism), amino acid metabolism (e.g., glycine, serine, and threonine metabolism), and energy-related processes (e.g., methane metabolism, other carbon fixation pathways) were enriched in leafy liverwort, suggesting an enhanced capacity for nutrient cycling and energy production. On the contrary, depletion of pathways related to biofilm formation, oxidative phosphorylation, and certain signaling pathways (e.g., FoxO and PI3K-Akt signaling pathways) in leafy liverworts might reflect adaptations to different microhabitat conditions or host physiological traits [28]. No significant enrichment was detected between different liverwort parts (upper vs. lower), suggesting that microbial functional potential remains relatively uniform across plant structures of a single liverwort type.

In conclusion, this study highlights the ecological significance of the associated microorganisms and their potential role in enhancing liverwort survival in Arctic ecosystems. Differences in microbial composition between lower and upper parts of liverworts, as well as between leafy and thalloid liverworts, suggest functional specialization. With this short report, we aim to encourage further investigations on a broader range of liverwort species to deepen our understanding of these microbial associations and their ecological impact.

## Supplementary Information

Below is the link to the electronic supplementary materials.

Supplementary file (XLSX 36.6 KB)

## Data Availability

No datasets were generated or analysed during the current study.
